# Psoriatic Arthritis Hospitalization Is Associated with Increased Health Care Charges: A Report from the National Inpatient Sample

**DOI:** 10.7759/cureus.12275

**Published:** 2020-12-25

**Authors:** Armaan Guraya, Eseosa J Sanwo, Karun M Nair, Sandhya Shri Kannayiram, Osahon N Idolor, Jesse O Odion

**Affiliations:** 1 Medicine, Midwestern University Chicago College of Osteopathic Medicine, Chicago, USA; 2 College of Medicine, University of Benin, Benin, NGA; 3 Internal Medicine, John H. Stroger, Jr. Hospital of Cook County, Chicago, USA; 4 Internal Medicine, University of Benin Teaching Hospital, Benin, NGA

**Keywords:** psoriasis, hospitalizations, health care outcomes, health care cost, dermatology, rheumatology, arthritis, mortality, average length of hospital stay, psoriatic arthritis

## Abstract

Background: This study aims to compare the outcomes of psoriasis hospitalizations with and without joint involvement. The primary outcome was inpatient mortality, while secondary outcomes were hospital length of stay (LOS) and total hospital charges.

Methods: Data were abstracted from the National Inpatient Sample (NIS) 2016 and 2017 databases. The NIS was searched for psoriasis hospitalizations with and without joint involvement as principal or secondary diagnosis using the International Classification of Diseases, tenth revision (ICD-10) codes. Psoriasis hospitalizations for adult patients (aged ≥18 years) from the above groups were identified. Multivariate logistic and linear regression analysis was used to adjust for confounders for the primary and secondary outcomes, respectively.

Results: There were over 71 million discharges included in the combined 2016 and 2017 NIS database. A total of 323,405 hospitalizations were for adult patients with either a principal or secondary ICD-10 code for psoriasis. Of these hospitalizations, 77,980 (24.11%) had joint involvement. Psoriasis hospitalizations with joint involvement had similar inpatient mortality (1.42% vs. 1.78%, adjusted odds ratio (AOR): 0.89, 95% CI: 0.76-1.05, p=0.159) compared with those without joint involvement. Psoriasis with joint involvement hospitalizations had a decrease in adjusted mean LOS of 0.15 days (95% CI: 0.26-0.04, p=0.007) compared with the group without joint involvement. Psoriasis with joint involvement hospitalizations had an increase in adjusted mean total hospital charges of $3,655 (95% CI: 2,146-5,164; p<0.0001) compared with the group without joint involvement.

Conclusions: Hospitalizations for psoriasis with and without joint involvement have similar inpatient mortality. However, joint involvement increases total hospital charges, which increases the burden to the health care system.

## Introduction

Psoriasis is a chronic, immune-mediated inflammatory skin disease affecting over seven million adults in the United States and is associated with substantial morbidity and mortality. It ranges in severity from a few scattered red, scaly plaques to involvement of almost the entire body surface. It may progressively worsen with age or wax and wane in its severity depending on genetic and environmental factors [1-3].

A systematic review found the prevalence of psoriasis in the United States to be 0.5% to 11.4% in adults and 0.1% to 1.4% in children [4]. Psoriasis with joint involvement, also known as psoriatic arthritis (PsA), is a complication of psoriasis resulting in chronic inflammation of synovial tissue, entheses, and skin, and is usually seronegative for rheumatoid factor [5].

A recent review of 20 epidemiologic studies found that the reported proportion of psoriatic arthritis among psoriasis patients in the United States ranges from 7% to 26% [6], Europe with 0.02%-0.42%, Japan is approximately 0.001%, and China is 0.02% [7-12].

There is a scarcity of studies on the hospitalization of psoriasis patients with and without joint involvement. This study aims to compare the outcomes of psoriatic arthritis hospitalizations to those of psoriasis without joint involvement. We aim to fill this knowledge gap in the literature.

## Materials and methods

Data source

The National Inpatient Sample (NIS) was searched for psoriasis hospitalizations with and without joint involvement as principal or secondary diagnosis using the International Classification of Diseases, tenth revision (ICD-10) codes. NIS is the largest hospitalization database in the United States [13-16]. NIS is a 20% probability sampling of different strata, structured to be representative at the national level [17-20]. Since patient data in NIS is de-identified, and the NIS database is available to the public, this study was exempted from institutional board review.

Inclusion criteria

We included all hospitalizations for patients aged 18 years and above. We used ICD-10 codes to identify principal/secondary diagnoses. Psoriasis hospitalizations were obtained by using the “L40” ICD-10 code. Psoriasis with joint involvement hospitalizations was obtained by using the “L405” ICD-10 code, while psoriasis without joint involvement hospitalizations was obtained by excluding psoriasis with joint involvement from psoriasis hospitalizations. The supplementary table provides a complete list of ICD-10 codes used (see Appendices).

Outcomes

The primary outcome was inpatient mortality. Hospital length of stay (LOS) and total hospital charges were secondary outcomes of interest.

Statistical analyses

Analyses were performed using Stata software (statistics and data), version 16 (StataCorp LLC, College Station, USA). A univariate logistic regression analysis using all variables and co-morbidities was used to calculate unadjusted odds ratios (ORs) for the primary outcome. All variables with P-values <0.2 were included in a multivariate logistic regression model. P-values <0.05 were considered significant in the multivariate analysis. A literature review was used to select confounders. Charleston index was used to control for comorbidity complexity. A multivariate logistic and linear regression model with all variables and co-morbidities were used accordingly to adjust for confounders for the secondary outcomes.

## Results

There were over 71 million discharges included in the combined 2016 and 2017 NIS database. A total of 323,405 hospitalizations were for adult patients with either a principal or secondary ICD-10 code for psoriasis. Of these hospitalizations, 77,980 (24.11%) and 245,425 (75.89%) were with and without joint involvement, respectively. Psoriasis with joint involvement hospitalizations had more females, whites, privately insured, 4th quartile median expected income for ZIP Code, hypertension, hypothyroidism, obesity, and less Charleston comorbidity index score ≥3 compared to those without joint involvement (Table 1).

**Table 1:** Baseline characteristics of hospitalizations of psoriasis patients with and without joint involvement Abbreviations: MI, myocardial infarction; COPD, chronic obstructive pulmonary disease; DM, diabetes mellitus; CHF, chronic congestive heart failure, CKD: chronic kidney disease, median household income: median household income for patient’s ZIP Code

Variables	Psoriasis (n=323,405)		
	Without joint (n=245,425)	With Joint (n=77,980)	p-value
Mean age (years)	61.09	60.82	0.057
Female	47.55%	57.29%	<0.0001
Race			<0.0001
White	79.94%	88.32%	
Black	6.62%	3.00%	
Hispanic	7.83%	4.92%	
Asian	2.48%	1.39%	
Native Americans	0.61%	0.60%	
Others	2.52%	1.77%	
Charleston comorbidity index			<0.0001
0	24.29%	27.03%	
1	22.65%	24.07%	
2	17.24%	17.64%	
≥3	35.82%	31.26%	
Hospital bed size			0.5263
Small	20.25%	20.60%	
Medium	28.56%	27.99%	
Large	51.19%	51.41%	
Hospital teaching status			0.7114
Nonteaching	31.83%	31.63%	
Teaching	68.17%	68.37%	
Hospital location			0.1498
Rural	8.46%	8.06%	
Urban	91.54%	91.94%	
Expected primary payer			<0.0001
Medicare	53.75%	53.29%	
Medicaid	14.79%	9.38%	
Private	28.25%	35.44%	
Self-pay	3.21%	1.89%	
Median household income (quartile)			<0.0001
1^st ^(0-25^th^)	25.84%	21.60%	
2^nd ^(26th-50^th^)	25.80%	25.08%	
3^rd^ (51st-75^th^)	25.16%	26.48%	
4^th^ (76th-100^th^)	23.20%	26.83%	
Hospital region			<0.0001
Northeast	22.60%	22.38%	
Midwest	24.90%	23.90%	
South	32.06%	35.14%	
West	20.43%	18.57%	
Dyslipidemia	42.02%	40.99%	0.0271
Old MI	6.75%	5.73%	<0.0001
Atrial fibrillation/flutter	10.87%	10.58%	0.301
COPD	19.83%	15.65%	<0.0001
Carotid artery disease	1.33%	1.25%	0.4328
Old stroke	6.60%	5.57%	<0.0001
Hypertension	43.83%	47.51%	<0.0001
Peripheral vessel disease	3.96%	3.17%	<0.0001
Hypothyroidism	14.83%	17.79%	<0.0001
DM type 1&2	32.34%	30.75%	0.0003
Obesity	24.05%	25.46%	0.0004
CHF	16.15%	13.05%	<0.0001
CKD	17.53%	15.56%	<0.0001
Liver disease	10.30%	8.62%	<0.0001
Maintenance hemodialysis	1.91%	1.19%	<0.0001
Smoking	29.92%	28.14%	<0.0001
Anemia	28.33%	28.78%	0.3068

In adult psoriasis hospitalizations, 5,485 ​​​​​​(1.70%) resulted in inpatient mortality. In psoriasis patients with joint involvement, 1,105 (1.42%) deaths occurred vs. 4,380 (1.78%) in the group without joint involvement (p=0.0019). The adjusted odds ratio (AOR) for inpatient mortality for psoriasis with joint involvement compared to without joint involvement was 0.89 (95% CI: 0.76-1.05, p=0.159). Psoriasis with joint involvement hospitalizations had a mean decrease in adjusted LOS of 0.15 days (95% CI 0.26-0.04, p=0.007) compared to without joint involvement. Psoriasis with joint involvement hospitalizations had an increase in adjusted mean total hospital charges of $3,655 than without joint involvement (95% CI: 2,146-5,164, p<0.0001) (Table 2).

**Table 2:** Outcomes of psoriasis hospitalizations with and without joint involvement Abbreviations: LOS, hospital length of stay; CI, confidence interval; USD, United States Dollars

	Psoriasis with joint involvement	Psoriasis without joint involvement	Adjusted Odds Ratio (AOR)	p-value
	% (95% CI)	% (95% CI)	(95% CI)	
Primary outcome				
In-hospital mortality	1.42	1.79	0.89 (0.76-1.05)	0.159
Secondary outcomes				
			Adjusted mean difference	
LOS, mean, days	4.86	5.25	0.15 (0.26-0.04)	0.007
Total charge, mean, USD	57,850	55,724	3,655 (2,146-5,164),	<0.0001

## Discussion

The major findings of our study are (i) total hospital charges in hospitalizations of psoriatic arthritis patients increased compared with psoriasis patients without arthritis, (ii) psoriatic arthritis patients had more cardiovascular (CV) comorbidities such as hypertension and obesity and were more likely to be above the 50th percentile for expected income compared with psoriasis patients without arthritis, (iii) psoriatic arthritis hospitalizations had similar inpatient mortality and decreased LOS compared with psoriasis without arthritis.

One study found that 21.4% of psoriasis patients do not see a specialist due to high costs; this underlines the importance of health care costs [21]. Our study found greater hospitalization expenses incurred by psoriasis patients with joint involvement than without. Fifteen percent of patients with psoriasis have undiagnosed psoriatic arthritis, a condition difficult to identify given its similarities to osteoarthritis, rheumatoid arthritis, and crystal arthropathy [22]. The presence of joint involvement further complicates the treatment because it requires the use of disease-modifying anti-rheumatic drugs or more novel therapies [23]. The combination of lack of longitudinal care and challenges in diagnosis and management reduce outpatient psoriatic arthritis treatment, potentially leading to more hospitalizations as the burden of their disease increases and perpetuating a cycle that burdens the healthcare system. Early intervention and collaboration by rheumatologists and dermatologists may, therefore, improve the outcomes of these patients. The optimum method for decreasing hospital charges in psoriatic arthritis would be to decrease admissions. This is important when the intersection of disease severity and patient socioeconomic status is considered. For instance, family incomes of patients with psoriasis correlate negatively with disease severity, and this severity is higher with family incomes below the social minimum [24]. However, in our study, more psoriatic arthritis patients were above the 50th percentile for expected income than psoriasis patients without arthritis.

Our study found that psoriatic arthritis hospitalizations had similar inpatient mortality compared with psoriasis without arthritis. Psoriatic arthritis hospitalizations had a statistically significant decreased adjusted LOS of 0.15 days compared with psoriasis without arthritis; however, this is not clinically significant.

In our study, psoriatic arthritis patients had more CV risk factors such as hypertension and obesity than psoriasis patients without joint involvement. Psoriatic arthritis is known to be associated with subclinical atherosclerosis after adjusting for traditional cardiovascular risk factors [25]. One study suggests that people with psoriasis with comorbidities, including CV, are more likely to have higher rates of urgent care visits, hospitalization, outpatient visits and incur greater costs than patients with psoriasis without such comorbidities [21]. Proper holistic treatment, which includes an emphasis on screening and preventative medicine, in these patients is essential considering these comorbidities are known to have far-reaching effects and mortality. The CV co-morbidities of hospitalized psoriatic patients should prompt further investigation into proper management and optimization of outcomes through a multidisciplinary approach.

This study has some limitations. (i) NIS contains claims data rather than clinical data [26]. Claims databases are administrative databases created for the purpose of billing. (ii) The severity of the disease is not graded by ICD-10 codes [27]; hence we cannot discern if psoriasis disease severity may have had affected the outcomes. (iii) Hospitalization data, and not individual patient data, is reported in NIS studies [28]. (iv) Immunosuppressant compliance information is not available in NIS [29]. (v) NIS does not contain information on the duration of illness [30].

## Conclusions

Hospitalizations for psoriasis with joint involvement had similar inpatient mortality compared with those without joint involvement. Psoriasis with joint involvement hospitalizations had a statistically significant, but clinically insignificant, decrease in LOS than without joint involvement. Psoriasis with joint involvement increases total hospital charges, which increases the burden to the health care system. Collaboration between the dermatologist and rheumatologist is needed to optimize outcomes.

## Appendices

**Table 3:** Supplementary table showing ICD-10 codes used in the study Abbreviations: MI, myocardial infarction; ICD-10, International Classification of Diseases, tenth revision.

	ICD-10 codes
Diagnosis codes	
Psoriasis	L40
Psoriasis with joint involvement	L405
Comorbidities	
Dyslipidemia	E78
Old MI	I252
Atrial fibrillation/flutter	I48
Chronic obstructive pulmonary disease	J41, J42, J43, J44
Carotid artery disease	I652
Old stroke	I63
Hypertension	I10
Peripheral vascular disease	I739
Hypothyroidism	E03
Diabetes mellitus Type 1&2	E10, E11
Obesity	E660, E6601, E6609, E661, E662, E668, E669
Congestive heart failure	I50
Chronic kidney disease	N18
Liver disease	K70, K71, K72, K73, K74, K75, K76, K77
Maintenance dialysis	Z992
Smoking	Z87891, F17200
Anemia	D50, D51, D52, D53, D55, D56, D57, D58, D59, D60, D61, D62, D63, D64
